# Imaging and MR Spectroscopy in Papillary Craniopharyngioma

**DOI:** 10.15388/Amed.2024.31.1.10

**Published:** 2024-02-27

**Authors:** Ashima Mahajan, Bheru Dan Charan, Sushant Agarwal, Vinay Goel, Leve Joseph Devarajan Sebastian, Vaishali Suri, Ashish Suri, Ajay Garg

**Affiliations:** 1 Department of Neuroimaging & Interventional Neuroradiology, All India Institute of Medical Sciences, New Delhi, India; 2 Department of Pathology, All India Institute of Medical Sciences, New Delhi, India; 3 Department of Neurosurgery, All India Institute of Medical Sciences, New Delhi, India

**Keywords:** Craniopharyngioma, Spectroscopy, Suprasellar, kraniofaringioma, spektroskopija, Suprasellar

## Abstract

Sellar-suprasellar masses exhibit a diverse range of differential diagnoses and it is feasible to establish a preliminary diagnosis before surgery by evaluating conventional CT scans and contrast-enhanced MRI results. Nevertheless, certain cases may present with inconclusive findings, making it challenging to anticipate the underlying tissue composition accurately through imaging alone. Researchers have explored the application of Proton MR spectroscopy in analyzing suprasellar tumors, and their investigations have revealed that it can complement traditional MRI by enhancing the accuracy of preoperative diagnoses. In this context, we report three biopsy-proven cases of suprasellar papillary craniopharyngioma where the MRS spectra derived from the solid component exhibited noticeable lipid peaks alongside reduced levels of choline and NAA. These findings played a pivotal role in facilitating the correct preoperative identification of papillary craniopharyngioma.

## Abstract (it)

Sellar-Suprasellar masės pasižymi įvairiomis diferencinėmis diagnozėmis, todėl prieš operaciją galima nustatyti preliminarią diagnozę, įvertinus įprastinės kompiuterinės tomografijos ir kontrastinės magnetinio rezonanso tomografijos rezultatus. Vis dėlto tam tikrais atvejais gali būti nevienareikšmės išvados, todėl sunku tiksliai numatyti pagrindinę audinių sudėtį remiantis vien tik vaizdiniais duomenimis. Mokslininkai nagrinėjo protonų MR spektroskopijos taikymą analizuojant viršugalvio navikus ir jų tyrimai atskleidė, kad ji gali papildyti tradicinį MRT, padidindama priešoperacinės diagnozės tikslumą. Atsižvelgdami į tai, aprašome tris biopsijos metu įrodytus supraselinės papilinės kraniofaringiomos atvejus, kai MRS duomenyse, gautuose iš kietojo komponento, buvo pastebimos lipidų smailės kartu su sumažėjusiu cholino ir NAA kiekiu. Šie duomenys buvo labai svarbūs, nes palengvino teisingą priešoperacinį papilinės kraniofaringiomos nustatymą.

## Introduction

Craniopharyngiomas are benign, mixed solid-cystic sellar/suprasellar masses arising from epithelial remnants of Rathke’s pouch. They account for nearly 3% of all intracranial tumors and are the most common nonglial brain tumors in children [1] Craniopharyngiomas often lead to a gradual decline in visual acuity due to anatomical distortions affecting the optic chiasm. It is beneficial to use radiology to distinguish between the adamantinomatous and papillary types since their prognoses differ. Only a few articles [2] have described the imaging differential between two entities based on age, MR signal intensity, calcification, and cyst formation. However, in some cases, imaging findings may remain equivocal, where advanced MR imaging tools like MRS may aid in making a correct diagnosis. The possible role of MRS in differentiating suprasellar mass has been described in the literature [3] but poorly described in papillary craniopharyngioma. In this article, we have described the MR imaging and MR spectroscopy characteristics in papillary craniopharyngioma.

**Material and method**. We retrospectively reviewed our radiographic exam and medical records from Jan 2022 to Jan 2023 in the Department of Neuroradiology AIIMS, New Delhi. We found three biopsy-proven cases of papillary craniopharyngioma. Two neuroradiologists independently assessed the CT and MRI findings of these cases. All patients have preoperative CT and MRI imaging, intraoperative and histopathological findings. The standard MRI studies (3T, Philips Ingenia) consisted of 3DT1, 3TT2, FLAIR, 3T1FS+C, and MR spectroscopy was obtained. A rectangular 1H-MRS voxel was positioned over the solid portion of the tumor projection, to avoid its cystic and necrotic regions. Additionally, precautions were taken to prevent contamination from the sinus, skull, and cerebral ventricles. Acquisition parameter (size of voxel=15x15 mm or 20x20 mm, TR: 2000 ms, TE: 35 ms, 128–256) suppression pulse outside the voxel, shimming and water suppression were done.

## Case illustration

### CASE 1

**Fig 1:**
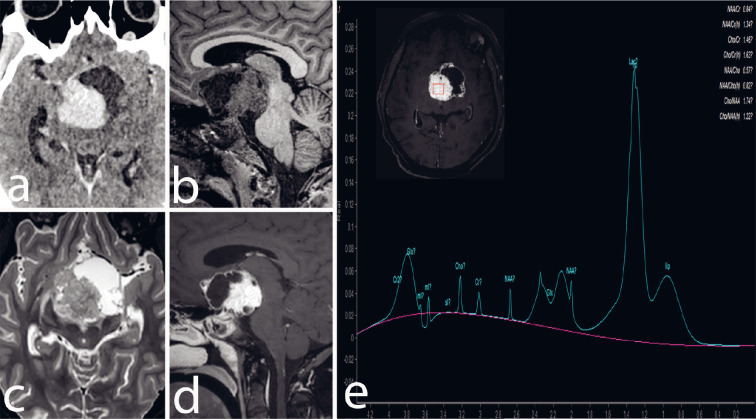
Contrast-enhanced CT scan (a), sagittal T1-WI (b), and axial T2-WI (c) show solid-cystic suprasellar mass with posterior extension into the interpeduncular cistern. The ventral cystic portion is hypointense on T1-WI and hyperintense on T2-WI, while the dorsal solid portion is isointense to grey matter in both T1- and T2-WIs, and enhances intensely in CECT (a) and sagittal T1 fat-sat post-contrast image (d). The optic chiasma is not seen separately from the lesion. T2 hyperintensity is seen in the right optic tract on the right side (1c). Proton MR spectroscopy at TE of 35 msec (e) from the solid component of the lesion shows a prominent lipid peak and reduced choline, creatinine, and NAA, corresponding to the Chernov type III(b) spectrum.

**Fig 2:**
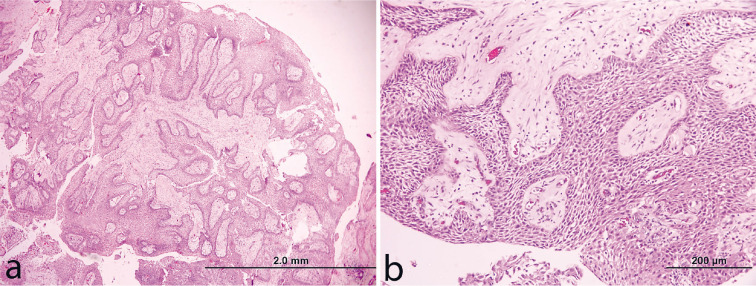
Hematoxylin and eosin stained sections (a & b) from the tumor show classical histomorphological features of a papillary craniopharyngioma comprising sheets of well-differentiated nonkeratinizing squamous epithelium arranged in papillary configurations around hyalinized fibrovascular cores

A 22-year-old man presented with a history of headaches persisting for four months and a gradual loss of vision, more pronounced on the right side, over the past two weeks. NCCT Brain did not reveal any calcifications. Contrast-enhanced brain CT (Fig. 1a) and MRI scans revealed a lobulated, mixed solid-cystic mass in the suprasellar region. This mass was expanding the optic chiasm (Fig. 1C) and T2 hyperintense signal intensity was noted in the right optic tract due to the mass effect (Fig. 1C). The cystic part of the mass displayed increased signal intensity on FLAIR imaging and had a rim of enhanced tissue along its cyst walls (Fig. 1d). The solid portion of the mass had signal characteristics similar to the brain cortex on both T1 and T2 imaging and exhibited intense enhancement after contrast injection (Fig. 1d). There were no signs of restricted diffusion in the mass. The pituitary gland was visibly separate from the lesion. The lesion extended into the interpeduncular cistern posteroinferiorly, causing splaying and compression of the midbrain. The midbrain and basal ganglia are pushed superiorly. There was compression of the anterior third ventricle withydrocephalus. Additionally, FLAIR imaging indicated elevated signal intensity, suggesting oedema along both optic tracts. Magnetic resonance spectroscopy (MRS) with a TE (echo time) of 35 msec showed a significant lipid peak in the solid part of the mass, along with reduced levels of NAA and choline, consistent with a Chernov’s type III B spectrum (Fig. 1e). The patient underwent a surgical procedure involving a left pterional craniotomy and subtotal excision of the mass. Subsequent histopathological examination confirmed the diagnosis of papillary craniopharyngioma, classified as WHO grade-1 (Fig. 2).

### CASE 2

**Fig 3:**
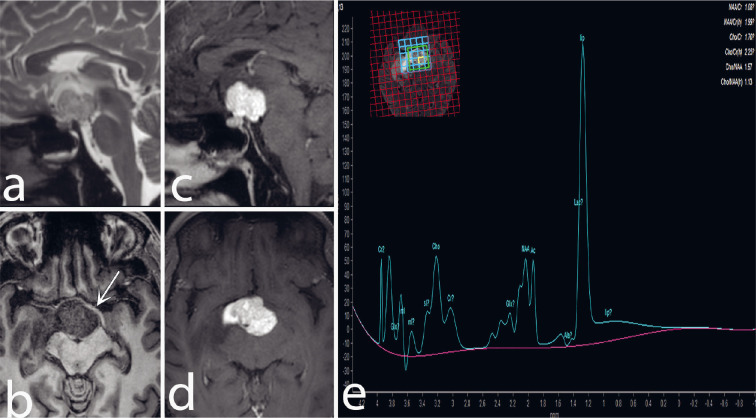
Sagittal T2-WI (a) and axial T1-WI (b) show a predominantly solid iso to mildly T2-hyperintense and T1-isointense suprasellar mass lesion; separate from optic chiasma (arrow in b) with associated oedema in the right temporal lobe. Post-gadolinium sagittal (c) and axial (d) T1 fat-sat images show intense enhancement in the lesion. Multivoxel Proton MR spectroscopy at 35 TE (e) shows a Chernov type III(b) spectrum with a prominent lipid peak and reduced choline, creatinine, and NAA.

A 32-year-old man complained of persistent headaches and a gradual, progressive loss of vision in his right eye spanning the past six months. MR Imaging showed a predominant solid extra-axial suprasellar mass lesion causing mass effect and oedema. The mass was iso-hyperintense on T2-WI and had intense enhancement following gadolinium administration (Fig. 3). MRS performed with echo times of 35 milliseconds, showed an elevated lipid peak within the mass with a reduction of other metabolites, suggesting a diagnosis of papillary craniopharyngioma. Right pterional craniotomy with right orbitotomy was done for gross tumor excision. Subsequent histopathological examination confirmed the diagnosis of papillary craniopharyngioma.

### CASE 3

**Fig 4:**
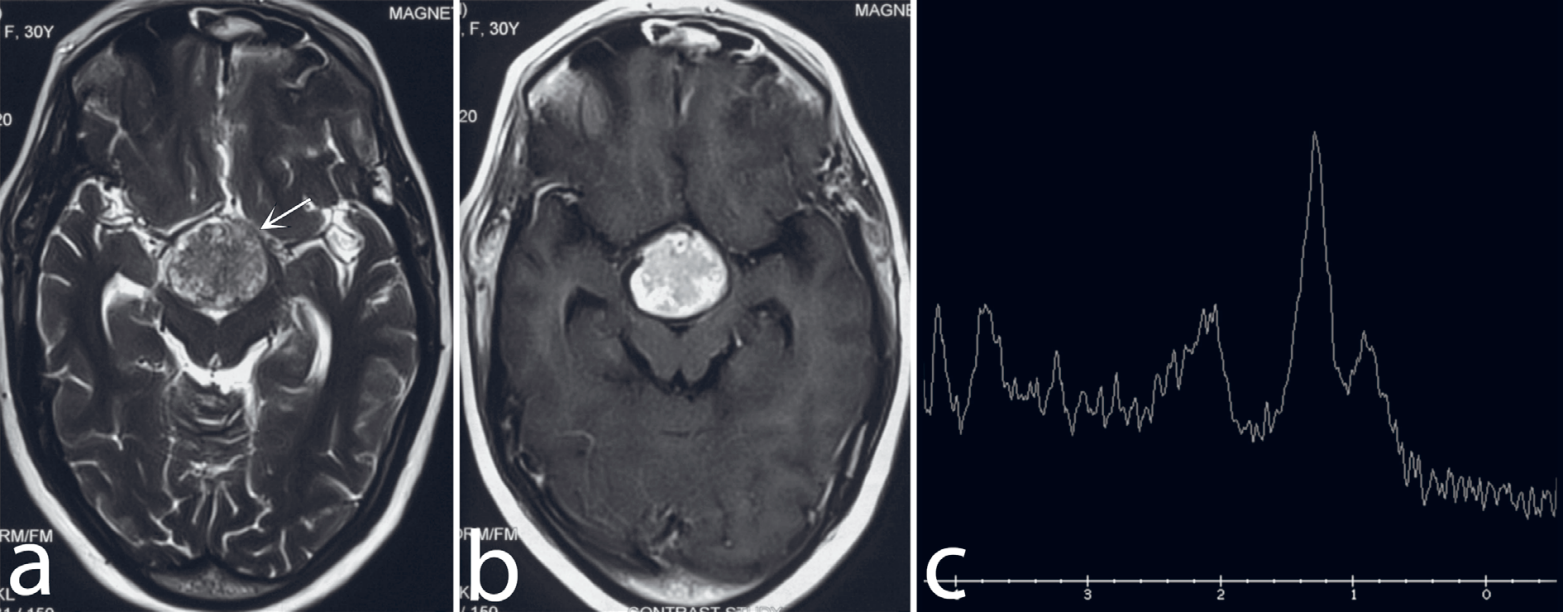
Axial T2-WI (a) shows a spherical, predominantly solid iso-hyperintense retrochiasmatic mass lesion separate from the chiasma (arrow in a). Post-gadolinium axial T1-WI (b) shows intense, solid, nearly homogeneous enhancement of the lesion. Single voxel Proton MRS at TE 144 (c) shows a prominent lipid peak at 1.3 ppm.

A 30-year-old lady presented with a gradual and progressive decline in vision in both eyes, with the right eye being more affected than the left over the past 15 months. She underwent surgery for a suprasellar mass 4 months ago. Histopathological examination confirmed it was papillary craniopharyngioma. A follow-up MRI revealed that a predominantly solid suprasellar mass remained, showing similar signal intensity to the surrounding tissue on T2-WI and intense enhancement on post-contrast T1-WI (Fig. 4). MRS conducted with a short echo time of 35 milliseconds indicated elevated lipid peaks alongside a decrease in other metabolites. Re-exploration of bifrontal craniotomy with gross tumor excision was done. Subsequent histopathological examination confirmed the diagnosis of papillary craniopharyngioma.

## Discussion

Two histological subtypes of craniopharyngiomas are recognized, the adamantinomatous and squamous-papillary, that are thought to arise from the epithelial remnants of Rathke’s pouch (adamantinomatous subtype) or the metaplasia of the squamous epithelial adenohypophyseal cells (papillary subtype) [4]. Typical conventional imaging findings have been described for both subtypes, with the adamantinomatous subtype being multilobulated, mixed solid-cystic lesions, showing T1 hyperintensity in the cystic component and 90% showing calcification and enhancement. Papillary craniopharyngiomas are predominantly solid, spherical enhancing masses that rarely calcify. When present, the cystic component of papillary craniopharyngiomas does not show the characteristic T1 hyperintensity shown by the adamantinomatous variant [5]. In our cases, MRI consistently revealed lesions that were either mixed solid-cystic or entirely solid in nature, with notable enhancement in the solid portion following the administration of a contrast agent. In instances where cystic components were present, they appeared as areas of decreased signal intensity on T1-WIs, and there was no calcification on CT scans. Furthermore, these cystic regions exhibited rim enhancement, strongly suggesting a papillary-type craniopharyngioma. Our case series imaging findings for papillary craniopharyngioma are compatible with the study done by Lee et al. [6]. Nevertheless, the invasion of the optic chiasm or the primary development of a craniopharyngioma within it is infrequent, with only a few reported cases in the medical literature [7–9]. The MRI findings associated with an intrachiasmatic craniopharyngioma have been described as a bilobed enlargement or a ‘pot belly’ expansion of the chiasm, characterized by its downward displacement and a crescent-moon shape observable in sagittal MRI images [7,8]. Notably, both of these distinct findings were evident in our particular case 1. Magnetic Resonance Spectroscopy (MRS) offers supplementary insights into the metabolic composition of brain tumors and can be particularly beneficial in cases where the diagnosis is uncertain. Chernov et al. [3,10] have established a classification system for the different pathological metabolic profiles observed in MRS spectra. This classification categorizes MRS spectra into types I, II, and III based on the dominant metabolite peak (NAA, choline, lipid) and further refines them into subtypes A, B, and C, taking into account the presence of lactate and lipid peak [10]. Sener et al. [1] conducted a study involving five patients with confirmed craniopharyngiomas and reported conspicuous peaks at 1–1.5 ppm in MRS, likely corresponding to lipid/cholesterol peaks. Their histological analysis confirmed elevated cholesterol levels in the cyst fluid, correlating with the MRS findings. In a study by Chernov et al. [3], which involved 40 patients with neoplasms in the suprasellar, hypothalamic, and third ventricle regions, including five craniopharyngiomas, 80% of the craniopharyngiomas exhibited a type IIIC MRS spectrum. This pattern was characterized by a significant reduction in major metabolites and the presence of multiple additional peaks, although it was suggested that this metabolic pattern might result from calcifications and microcysts within the tissue under investigation [3]. Jouibari et al. [11] examined 28 suprasellar tumours using both MRI and MRS. Among their findings, four out of five craniopharyngioma patients displayed prominent lipid peaks. This discovery led to a revised preoperative diagnosis in two cases. In contrast, other sellar tumors, such as optic pathway/hypothalamic gliomas (OCHGs), typically exhibit type II peaks on MRS, characterized by elevated choline levels, sometimes with the presence of lactate and [3,12] In our study, MRS consistently revealed a significant lipid peak (Type IIIB) alongside reduced levels of NAA and Choline within the lesions. Available literature describes lipid peaks in adamantinoma craniopharyngioma with doubtful MRS role papillary variant. These MRS findings reinforced our preoperative diagnosis of craniopharyngioma where the typical features like calcification are missing. Our results underscore the importance of utilizing MR Spectroscopy as a valuable advanced imaging tool for intracranial tumor assessment and diagnosis. Our study have few limitations, as we have a low number of cases, and a retrospective nature. More studies with a large number of cases are needed to study MRS in papillary craniopharyngioma.

## Conclusion

MR Spectroscopy plays a crucial role in the diagnostic process, serving as a valuable adjunct to contrast-enhanced MRI when assessing suprasellar tumors. It enhances diagnostic precision, particularly when conventional imaging results are inconclusive.

## Availability of data and material (data transparency)

Yes
